# Concentration of Petroleum-Hydrocarbon Contamination Shapes Fungal Endophytic Community Structure in Plant Roots

**DOI:** 10.3389/fmicb.2016.00685

**Published:** 2016-05-12

**Authors:** Guillaume Bourdel, Alice Roy-Bolduc, Marc St-Arnaud, Mohamed Hijri

**Affiliations:** Institut de Recherche en Biologie Végétale, Département de Sciences Biologiques, Université de Montréal, MontréalQC, Canada

**Keywords:** fungi, endophytes, petroleum-hydrocarbon contamination, pyrosequencing, ribosomal DNA, *Eleocharis erythropoda*, *Populus balsamifera L.*

## Abstract

Plant-root inhabiting fungi are a universal phenomenon found in all ecosystems where plants are able to grow, even in harsh environments. Interactions between fungi and plant roots can vary widely from mutualism to parasitism depending on many parameters. The role of fungal endophytes in phytoremediation of polluted sites, and characterization of the endophytic diversity and community assemblages in contaminated areas remain largely unexplored. In this study, we investigated the composition of endophytic fungal communities in the roots of two plant species growing spontaneously in petroleum-contaminated sedimentation basins of a former petro-chemical plant. The three adjacent basins showed a highly heterogeneous pattern of pollutant concentrations. We combined a culture-based isolation approach with the pyrosequencing of fungal ITS ribosomal DNA. We selected two species, *Eleocharis erythropoda* Steud. and *Populus balsamifera L*., and sampled three individuals of each species from each of three adjacent basins, each with a different concentration of petroleum hydrocarbons. We found that contamination level significantly shaped endophytic fungal diversity and community composition in *E. erythropoda*, with only 9.9% of these fungal Operational Taxonomic Units (OTUs) retrieved in all three basins. However, fungal community structure associated with *P. balsamifera* remained unaffected by the contamination level with 28.2% of fungal OTUs shared among all three basins. This could be explained by the smaller differences of pollutant concentrations in the soil around our set of *P. balsamifera* sampless compared to that around our set of *E. erythropoda samples*. Our culture-based approach allowed isolation of 11 and 30 fungal endophytic species from surface-sterilized roots of *E. erythropoda* and *P. balsamifera*, respectively. These isolates were ribotyped using ITS, and all were found in pyrosequensing datasets. Our results demonstrate that extreme levels of pollution reduce fungal diversity and shape community composition in *E. erythropoda*. Our findings shed light on the effect of soil petroleum contamination on fungal endophytic communities and could help to develop strategies for improving phytoremediation using fungal endophytes.

## Introduction

Phytoremediation uses plants to remove pollutants from contaminated soils (Cunningham and Berti, 1993). This decontamination method is cost-effective as well as environmentally friendly biotechnology compared to conventional methods (Salt et al., 1998; Pilon-Smits, 2005). Traditional approaches usually involve excavation and transportation of soils for *ex situ* chemical extraction of pollutants, an expensive and energy demanding technology, or disposal, a process known as *Dig and Dump* (Chaudhry et al., 2005; Göhre and Paszkowski, 2006; Kaimi et al., 2007). However, phytoremediation has several downsides, including a generally lower efficiency of pollutant removal, longer treatment and a depth of treated soils limited to the zone colonized by plant roots (Chaudhry et al., 2005; Pilon-Smits, 2005). Thus many attempts have been made to enhance phytoremediation efficiency by selecting high-performing plant species and cultivars, and their associated microorganisms, such as rhizospheric microbes, including symbiotic and endophytic microorganisms (Hassan et al., 2011, 2013; Bell et al., 2014).

Endophyte is defined literally as any organism living inside a plant (from Greek *endon* = inside and *phyton* = plant). The term endophyte usually refers to bacteria (Kobayashi and Palumbo, 2000) and fungi (Stone et al., 2000), but algae (Peters, 1991), and insects (Feller, 1995) can also be endophytes. Endophytic bacteria and fungi have been found in all plants studied so far, providing examples of the broad variety of colonizing organisms as well as hosts for endophytic interactions (Schulz and Boyle, 2005; Hyde and Soytong, 2008). There are many different types of interactions between endophytic organisms and plants spanning from mutualism (Sieber, 2002) to parasitism (Kogel et al., 2006; Paszkowski, 2006). Plants may benefit from the presence of endophytes such as increased growth (Petrini, 1991; Varma et al., 1999; Schulz et al., 2006; Rodriguez et al., 2009), and resistance against biotic (Bacon et al., 1977; Carroll, 1988; Melo et al., 2009) and abiotic stresses, e.g., drought tolerance (Schardl et al., 2004; Hamilton and Bauerle, 2012).

The biodiversity of endophytic organisms has been increasingly investigated in recent decades, and methods for studying endophytes within plants have evolved during that time. Endophytic detection was first carried out using plant tissue staining and microscopy (Jumpponen and Trappe, 1998; Jumpponen, 2001; Porras-Alfaro and Bayman, 2011). Isolation of endophytes using surface-sterilized plant tissues on growth media has been intensively used for bacteria and fungi (Schulz and Boyle, 2005; Schulz et al., 2006). These culture-dependent methods have been shown to provide better results than microscopic observation, although they require considerable effort and time, and can also be biased depending on the type of media used or sampling effort (Hyde and Soytong, 2008; Stefani et al., 2015). In recent years, the study of endophytes has shifted towards culture-independent methods using molecular approaches such as PCR, cloning and sequencing, and next-generation sequencing (Ganley et al., 2008). These molecular approaches can be directly applied to any plant tissue and have proven useful to study changes in community structure.

Despite the increasing number of studies published on endophytes, interactions between fungal endophytic organisms and their hosts are still poorly understood, particularly in the context of phytoremediation (Soleimani et al., 2010). Furthermore, bacterial endophytes have traditionally received more attention, and been more intensively studied in a wider range of contaminant degradations than fungal endophytes (reviewed in Rylott, 2014). While the role of fungi in plant growth-promotion remains nearly unexplored in phytoremediation (Li et al., 2012), some authors have used bacterial and fungal consortia to enhance phytoremediation using poplar (Knoth et al., 2014). It has been shown that the use of endophytic fungi enhances phytoremediation applications due to fungal developmental plasticity and fungal production of a variety of secondary metabolites and enzymes that can degrade or sequester pollutants (Tan and Zou, 2001; Schulz et al., 2002; Hyde and Soytong, 2008). Soleimani et al. (2010) have studied the effect of two-grass species *Festuca arundinacea* Schreb. and *Festuca pratensis* Huds. inoculated with endophytic fungi *Neotyphodium coenophialum* and *Neotyphodium uncinatum*, respectively, on the degradation of petroleum hydrocarbons in a contaminated soil. Although the authors used plants and endophytic strains that are not native to the contaminated site and the fungi used for inoculation belong to the *Balansia* group, a group of well-studied endophytic fungi known for their beneficial roles for grasses (Bacon et al., 1977; Schulz et al., 1993), they found that inoculated plants were able to decontaminate soils better than non-inoculated plants (Soleimani et al., 2010).

The aim of this study is to investigate the community structure of endophytic fungi in roots of *Eleocharis erythropoda* Steud. and *Populus balsamifera* L., two plant species growing spontaneously in petroleum-contaminated basins of a former petrochemical plant. These plant species have been selected for this study because they were abundant at the sampling site and can be found in three adjacent basins where concentrations of petroleum hydrocarbon pollutants varied from no-contamination to low or extreme contamination. For both species, three individual plants were sampled from each basin. We characterized endophytic fungal communities associated with the roots of the two species using 454 sequencing of the ITS region and evaluated the changes in community composition in response to contaminants concentrations. Fungi were also isolated and culture-dependent community structures were compared to those obtained with amplicon sequencing.

## Materials and Methods

### Site Description and Sampling

The sampling site, a former petrochemical plant located near Montreal (Varennes, 45°43 N, 73°22 W, Montreal, QC, Canada), consists of three sedimentation basins separated by dikes in which petroleum hydrocarbon wastes were discarded for many decades. A previous study conducted in one of these three basins has shown that the site was characterized by patchy distribution of spontaneous vegetation mainly dominated by *Eleocharis obtusa* Willd. and *Panicum capillare* L. (Desjardins et al., 2014). In this study, we chose to sample the roots of *E. erythropoda* Steud. and *P. balsamifera* L., because they were found in the three basins although they were not the dominant plant species. We collected three replicates of each plant species in each of the three basins, i.e., 18 root systems sampled. Individual plants were collected with the soil surrounding the root system. For each individual plant, the complete root system was cut and rhizospheric soil was removed and kept. Roots of each individual plant were cleaned and cut in small pieces that were used for DNA extraction and for isolation of endophytes. Composite rhizospheric soil samples were also prepared for each plant species and each basin. Chemical analyses for assessing petroleum hydrocarbon contaminant concentrations were performed on composite samples for each basin by mixing rhizospheric soil of *E. erythropoda* and *P. balsamifera* individuals collected within the same basin resulting in three composite samples for each plant species.

### Soil Contamination Analysis

Soil analyses were performed by a commercial service provided by Maxxam Analytics (Montreal, QC, Canada). We measured polycyclic aromatic hydrocarbons (PAHs), aliphatic hydrocarbons C10–C50 and polychlorinated biphenyl (PCB). Soil analysis data are shown in **Table 1.** Analysis revealed concentrations of 3000, 41000, and 91000 mg⋅kg^-1^ of total petroleum hydrocarbon (TPH) in soil where the three individual plants of *E. erythropoda* were sampled, and concentrations of 280, 380, and 9040 mg⋅kg^-1^ of TPH where the three individual pants of *P. balsamifera* were sampled.

**Table 1:** Concentrations of petroleum hydrocarbon pollutants (polycyclic aromatic hydrocarbons (PAHs) (Chaudhry et al., 2005), alkanes [C10–C50] and total polychlorinated biphenyls [PBCs]) in the sediments of the three basins where *Eleocharis erythropoda* (E) and *Populus balsamifera* (P) plants were collected.

Petroleum Hydrocarbons			Accepted limit values^1^	Concentrations (mg.kg^-1^)					
				
				Basin 1		Basin 2		Basin 3	
						
				*E*	*P*	*E*	*P*	*E*	*P*
90 Polycyclic Aromatic Hydrocarbons (PAHs)
		Acenaphthene	**100**	**760**	**150**	**620**	<0.1	0.2	<0.1
		Anthracene	**100**	**340**	28	**570**	3.8	6.7	3.7
		Benzo(a)anthracene	**10**	21	7.2	**83**	0.7	1.5	<0.1
		Benzo(c)phenanthrene	**10**	9	3	**39**	0.3	1.3	<0.1
		Chrysene	**10**	**23**	8.8	**99**	1.2	1.1	<0.2
		Fluoranthene	**100**	97	30	**300**	0.5	2.8	<0.1
		Fluorene	**100**	**710**	78	**630**	0.2	0.3	0.1
		Naphtalene	**50**	11	**52**	**150**	<0.1	<0.1	<0.1
		Phenanthrene	**50**	**2700**	**357**	**4300**	<0.1	1	0.2
		Pyrene	**100**	**150**	47	**440**	1.9	14	0.1
		2-Methylnaphthalene	**10**	**160**	**62**	**240**	<0.1	<0.1	<0.1
		1-Methylnaphthalene	**10**	**320**	**77**	**300**	<0.1	<0.1	<0.1
		1,3-Dimethylnaphthalene	**10**	**390**	**123**	**580**	<0.1	<0.1	<0.1
		2,3,5-Trimethylnaphthalene	**10**	**150**	**18**	**280**	<0.1	0.2	<0.1

**Alkanes (C10–C50)**			**3500**	**41000**	9040	**91000**	380	3000	280
**Polychlorinated Biphenyls**			**10**	<1	<1	**20**	<0.01	0.3	0.1


*^1^Values corresponding to the maximum limit accepted for contamination of industrial areas by the Sustainable Development, Environment, Wildlife, and Parks Department of the Province of Québec, Montreal, QC, Canada. (http://www.mddefp.gouv.qc.ca/sol/terrains/politique/annexe_2_tableau_1.htm). Concentrations (mg.kg^-1^) above the accepted limits are in bold.*

### Endophytic Fungal Isolation Procedure

*Eleocharis erythropoda* and *P. balsamifera* root samples were used to isolate fungal endophytes. Root samples were processed immediately after their collection from the site. Roots were cleaned using tap water in order to remove soil particles and were put in 50 mL Falcon tubes. After cleaning, roots were surface-sterilized using the following procedure: roots were first washed three times in sterile water, followed by immersion in 3% H_2_O_2_ solution for 4 min, 6% NaOCl solution for 7.5 min, 96% ethanol for 2.5 min and 3% H_2_O_2_ solution for 3.5 min. Roots were rinsed three times between treatments using autoclaved water and were rinsed again five times at the end of the disinfection procedure.

The isolation media used were 0.1 × strength PDA medium (Difco), 0.1 × Malt medium (Difco) and Gel-Gro^®^ medium containing 0.4% Gel-Gro^®^ (ICN Biochemicals Aurora, OH, USA) and 0.03% MgSO_4_ (Silvani et al., 2008). Streptomycin (100 μg⋅μL^-1^) was added to each medium to limit bacterial growth. The efficiency of surface sterilization was assessed by following up the growth for potential contamination of microorganisms in our media using both last rinsing water and root-imprints. A 150 μL aliquot of the last rinsing water was plated on each medium in triplicate and incubated at 27°C for 4 weeks to assess for the presence of living microorganisms. Root-imprints on plates containing each media type were also used to test for the presence of microorganisms living on the surface of the roots. To isolate endophytes, roots were cut in 5 mm segments, and for each plant sample from each contaminated basin, 10 root segments were plated, with 5 segments per Petri dish on each of the three isolation media. For the Gel-Gro^®^ medium, root segments were placed inside a 150 μL drop of medium as described in Silvani et al. (2008). Plates were incubated at 27°C and checked after 5 days for fungal growth emerging from the root fragment ends, under a dissecting microscope. Fragments showing growing hyphae were transferred on 0.1% of Malt medium and incubated at 27°C with a single fragment per plate as described by Silvani et al. (2008). Fungal isolates were further subcultured in triplicates using the same culture media used for isolation. DNA extraction, PCR amplification, and sequencing of isolated fungal endophytes.

Pure fungal isolates were grown on liquid 0.1% of Malt medium and used for DNA extraction, which was performed using the E.Z.N.A.^®^ Forensic DNA Kit (Omega Bio-Tek, Norcross, GA, USA) according the manufacturer’s instructions with slight modifications: fungal mycelia were first dried out on clean paper towel to remove liquid medium, heated at 50°C in 1.5 mL tubes and then crushed with liquid nitrogen using a micro pestle. No DTT buffer was used in the DNA extraction, the incubation time at step 5 of the protocol was changed to 60 min, and the final elution volume used was 70 μL.

PCR amplification of ITS regions was performed using ITS1 (5′-TCCGTAGGTGAACCTGCGG-3′) and ITS4 (5′-TCCTCCGCTTATTGATATGC-3′) primers (White et al., 1990). PCR reactions were performed in 50 μL volume containing 2 μL of DNA template, 1 × PCR Buffer, 0.4 μM of each primer, 0.25 mM of dNTPs, 1 μg⋅μL^-1^ of bovine serum albumin, 2.5 mM of MgCl_2_, 1 U of HotStar *Taq* polymerase (QIAGEN, Toronto, ON, Canada) or 0.75 U of Kapa *Taq* polymerase (VWR, Montreal, QC, Canada). Cycling conditions were as follows: 15 min for HotStar *Taq* or 2 min for Kapa *Taq* at 95°C, 32 cycles of 94°C for 30 s, 55°C for 1 min and 72°C for 1 min, followed by a final elongation step of 72°C for 10 min. PCR reactions were run on a MaterCycler Pro S thermocycler (Eppendorf, Mississauga, ON, Canada). PCR patterns were revealed with agarose gel electrophoresis using GelRed (Biotium, Hayward, CA, USA). Positive PCR reactions were directly sequenced at the Genome Quebec Innovation Centre (McGill University, Montreal, QC). Geneious Pro v.8.1.8 (Drummond et al., 2009) was used to analyze sequences. Blast nucleotide searches were performed at the NCBI web site^1^ Nucleotide sequences of ITS were deposited in GenBank under the accession numbers: KU179236-KU179276.

### 454-Amplicon Sequencing

DNA of surface sterilized roots was extracted using the DNeasy Plant Mini Kit (QIAGEN, Toronto, ON, Canada) according the manufacturer’s instructions. Two hundred micro gram of roots were crushed using a mortar and pestle in liquid nitrogen. The complete ITS region was amplified using the primers ITS1F (5′-CTTGGTCATTTAGAGGAAGTAA-3′) (Gardes and Bruns, 1993) and ITS4 (5′-TCCTCCGCTTATTGATATGC-3′) (White et al., 1990). Forward primers for each sample were tagged with unique multiplex identifiers (MIDs). PCR amplifications were done in a volume of 20 μL containing 0.275 μM of each primer, 415 ng⋅μL^-1^ of bovine serum albumin, 0.83 μL of Tween 20 1% solution (v/v), 0.83 μL of dimethyl sulfoxide 0.275 mM of dNTPs, final concentration of 2.75 mM of MgCl_2_, 1 × of *Taq* polymerase buffer, 0.5 U of *Taq* polymerase (QIAGEN, Toronto, ON, Canada) and 2 μL of genomic DNA as a template. Amplicons were purified using the QIAquick PCR Purification Kit (QIAGEN, Toronto, ON, Canada). Triplicate samples were pooled together and DNA concentration was quantified using the Quant-iT PicoGreen dsDNA assay kit (Life Technologies, Burlington, ON, Canada). All samples were then pooled together in an equimolar ratio and were sent to sequencing at the Genome Quebec Innovation Centre (McGill University, Montréal, QC, Canada) using the 454 GS-FLX Titanium Lib-L chemistry (Roche, Laval, QC, Canada).

### Sequence Analysis

Sequences were pre-processed in Mothur v1.29.2 (Schloss et al., 2009) and then imported in Qiime (Caporaso et al., 2010) for quality filtering. Low quality ends were trimmed using a moving window of 50 bp with a minimum quality score of 25. Reads shorter than 200 bp, longer than 1000 bp, with more than two ambiguities (Ns), with homopolymers longer than 8 bp, or with two or more mismatches in the primer or barcode region were eliminated. Reads were then pruned to a fixed length (400 bp) with Mothur. Usearch v7.0 (Edgar, 2010) was used for chimera checking and clustering into Operational Taxonomic Units (OTUs). Singletons (i.e., OTUs represented by only one read) were eliminated because they are mostly artifacts that can be attributed to sequencing errors (Tedersoo et al., 2010). Finally, reads were mapped back into an OTU table and all global singletons (i.e., OTUs represented by a single sequence in the complete dataset) were eliminated. Taxonomy was assigned to each OTU consensus sequence using the UNITE database (Kõljalg et al., 2013) in Mothur with a minimum bootstrap value of 60%. Raw sequences were deposited in the NCBI Sequence Read Archive and are available under the project number PRJNA272728.

### Statistical Analysis

All statistical analyses were conducted in R v.3.0.2^2^ (R Foundation for Statistical Computing). In order to find patterns in fungal endophytes diversity, a rarefaction analysis was computed with the ‘iNEXT’ package (Hsieh et al., 2013). Samples were rarefied at 1000 reads in order to compare richness. We also evaluated total richness with the Chao estimator (Chao, 1984). Sample coverage was computed as suggested by Chao and Jost (2012) for both rarefied and complete samples. ANOVA and Tukey HSD *post hoc* tests were done using the ‘aov’ and ‘TukeyHSD’ functions of the ‘stats’ package to test for significant differences in richness between contamination level and host plant species. A Principal coordinate analysis (PCoA) was used to explore the patterns of fungal OTU composition across TPH levels and host species. The Hellinger distance was computed using the ‘decostand’ and ‘vegdist’ function in ‘vegan’. This distance metric puts more emphasis on species proportions than on abundances (Ramette, 2007; Anderson et al., 2011) and is therefore appropriate in this case (number of reads not a direct measure of OTU abundance in the environment). A Permutational Multivariate Analysis of Variance was performed with the ‘adonis’ function in ‘vegan’ with 9999 permutations to check the significance of site grouping according to host species and contamination levels (plant and TPH effects). Then we performed post-hoc pairwise comparisons (for the different hosts within the root samples and within the soil samples only) and corrected the p-values for multiple comparisons with in Holm correction using the ‘p.adjust’ function in the ‘stats’ package. Finally, we described endophytic community variations in terms of taxonomy by computing the main number of reads for each major fungal classes.

## Results

### Culture-Based Isolation of Fungal Endophytes from Sterilized-Plant Roots

A total of 41 endophytic fungal isolates were recovered from *E. erythropoda* (11 isolates) and *P. balsamifera* (30 isolates) roots. Nine endophytes were isolated from *E. erythropoda* roots sampled in soils containing 3000 and 40000 mg⋅kg^-1^ TPH while only two strains were isolated from *E. erythropoda* roots sampled in the highest contamination level (91000 mg⋅kg^-1^ TPH). Twenty-three fungal strains were recovered from *P. balsamifera* roots sampled in soil containing 280–380 mg⋅kg^-1^ TPH and seven from the 9040 mg⋅kg^-1^ TPH concentration (**Table 2**). Sequencing of the ITS region revealed that *Fusarium* and *Leptosphaeria* were the most represented genera with five isolates belonging to each genus, which were both isolated from *P. balsamifera* roots. All the isolated endophytes belonged to Dikarya (Ascomycota and Basidiomycota), with 39 isolates being Ascomycota and only two isolates being Basidiomycota.

**Table 2:** List of isolated endophytic fungal isolates from surface-sterilized roots of *E. erythropoda* (E) and *P. balsamifera* (P).

Isolate	Plant	TPH	Identification	Closest match (accession No., % identity)
1	E	3	Unidentified fungus 01	Uncultured fungus (FM875862, 99%)
2	E	3	*Plectosphaerella* sp. 01	*Plectosphaerella cucumerina* (JX431888, 99%)
3	E	3	*Plectosphaerella* sp. 02	*Plectosphaerella cucumerina* (JX431888, 99%)
4	E	41	Pleosporaceae sp.	Uncultured Pleosporaceae (FM178248, 98%)
5	E	41	*Alternaria* sp.	*Alternaria rosae* (KC797650, 97%)
6	E	91	Unidentified fungus 02	Uncultured fungus (JQ989297, 99%)
7	E	91	*Emericellopsis* sp.	*Emericellopsis minima* (U57675, 100%)
8	E	3	Unidentified fungus 03	Uncultured fungus (JQ989297, 100%)
9	E	3	Unidentified fungus 04	Uncultured fungus (JQ989322, 99%)
10	E	3	Sordariales sp. 01	Uncultured Sordariales (JN802311, 98%)
11	E	3	Sordariales sp. 02	Uncultured Sordariales (JN802311, 97%)
12	P	9	*Fusarium oxysporum* 01	*Fusarium oxysporum* (JX045827, 99%)
13	P	9	Unidentified fungus 05	Fungal sp. (GU566228, 99%)
14	P	9	*Penicillium* sp. 01	*Penicillium* sp. (KF811430, 100%)
15	P	9	Pleosporales sp.	Pleosporales sp. (KC460810, 98%)
16	P	9	Unidentified fungus 06	Uncultured fungus (KF800645, 99%)
17	P	9	Ascomycota sp.	Ascomycota sp. (AB566314, 100%)
18	P	9	*Schizophyllum* sp.	*Schizophyllum commune* (JN882337, 97%)
19	P	0.3	Unidentified fungus 07	Fungal sp. (GU566255, 100%)
20	P	0.3	*Pythium* sp. 01	*Pythium sterilum* (JQ898474, 98%)
21	P	0.3	*Leptosphaeria* sp. 01	*Leptosphaeria* sp. (AB752252, 100%)
22	P	0.3	Unidentified fungus 08	Fungal sp. (GU566255, 100%)
23	P	0.3	*Pythium* sp. 02	*Pythium periplocum* (GU811234, 100%)
24	P	0.3	*Leptosphaeria* sp. 02	*Leptosphaeria* sp. (AB752252, 99%)
25	P	0.3	*Cadophora luteo-olivacea*	*C. luteo-olivacea* (GU128589, 100%)
26	P	0.3	Unidentified fungus 09	Fungal sp. (GU566294, 99%)
27	P	0.3	Leptosphaeria sp. 03	*Leptosphaeria* sp. (AB752252, 100%)
28	P	0.3	*Fusarium oxysporum* 02	*Fusarium oxysporum* (JX045827, 99%)
29	P	0.3	*Fusarium oxysporum* 03	*Fusarium oxysporum* (KC254033, 100%)
30	P	0.2	Unidentified fungus 10	Fungal sp. (GU566255, 100%)
31	P	0.2	*Rhodotorula* sp.	*Rhodotorula* sp. (AM160641, 98%)
32	P	0.2	Unidentified fungus 11	Fungal sp. (GU566255, 100%)
33	P	0.2	Unidentified fungus 12	Fungal sp. (GU566294, 100%)
34	P	0.2	*Fusarium oxysporum* 04	*Fusarium oxysporum* (KC202938, 99%)
35	P	0.2	*Fusarium oxysporum* 05	*Fusarium oxysporum* (HM179530, 100%)
36	P	0.2	*Penicillium* sp. 02	*Penicillium* sp. (HQ850367, 100%)
37	P	0.2	Unidentified fungus 13	Fungal sp. (GU566255, 100%)
38	P	0.2	*Penicillium* sp. 03	*Penicillium* sp. (HQ850367, 100%)
39	P	0.2	*Leptosphaeria* sp. 04	*Leptosphaeria* sp. (AB752252, 100%)
40	P	0.2	*Phoma* sp.	*Phoma herbarum* (JX077064, 99%)
41	P	0.2	*Leptosphaeria* sp. 05	*Leptosphaeria* sp. (AB752252, 99%)


*Total petroleum hydrocarbons (TPH) concentration is given in grams per Kg.*

### Endophyte Fungal Diversity and Community Composition

**FIGURE 1:**
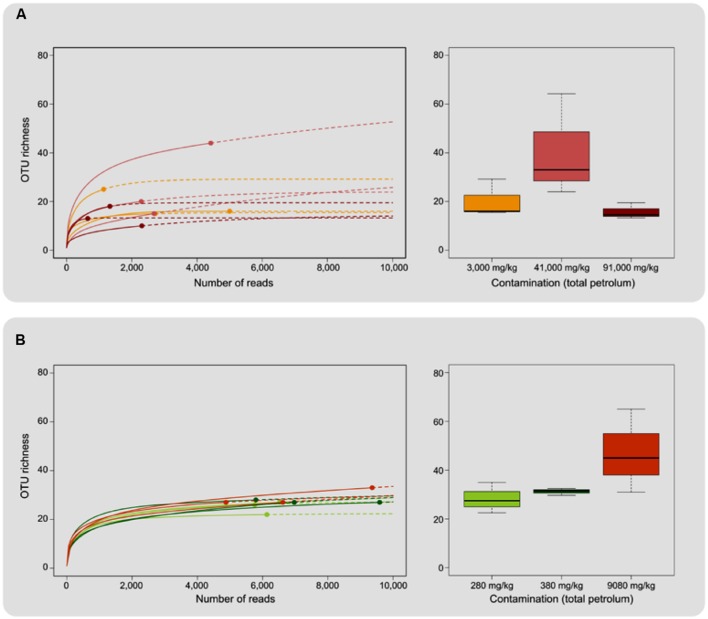
**Operational Taxonomic Unit richness associated with **(A)***Eleocharis erythropoda* and **(B)***Populus balsamifera* roots.** The left hand portion of the figure shows rarefaction curves, solid lines representing interpolation and dashed lines total richness extrapolation up to 10,000 reads. Right hand portion displays average total richness evaluated with the Chao estimator for each contamination level. Individual samples were colored based on the level of petroleum hydrocarbon contamination.

A total of 217,086 sequences were retrieved from pyrosequencing of the ITS region after quality filtering, which clustered into a total of 592 OTUs. The number of sequences per sample ranged from 647 to 9586, with an observed richness ranging from 10 to 44 OTUs (**Table 3**). All rarefaction curves were close to saturation and Good’s coverage was close to 1 (**Figure 1**; **Table 3**), indicating that sequencing depth was appropriate. ANOVA did not reveal significant differences in richness between contamination levels for either *E. erythropoda* or *P. balsamifera* in rarefied richness or Chao total richness estimators (**Figure 1**).

**Table 3:** Mean and standard error of the number of reads, richness and coverage of fungal OTUs recovered by 454 pyrosequencing.

	Number of reads		Observed OTU richness	Coverage	Total OTU richness (Chao estimator)	Rarefied OTU richness	Coverage at 1000 reads
***E. erythropoda***
3000 mg/kg		2952.67 ± 1120.58	18.67 ± 3.18	0.9984 ± 0.0014	20.22 ± 4.47	16.78 ± 3.79	0.9963 ± 0.0009
41000 mg/kg		3131.00 ± 654.75	26.33 ± 8.95	0.9980 ± 0.0001	40.41 ± 12.20	19.43 ± 6.09	0.9943 ± 0.0017
91000 mg/kg		2105.67 ± 403.87	13.67 ± 2.33	0.9983 ± 0.0003	15.75 ± 1.91	12.69 ± 2.66	0.9980 ± 0.0012
***P. balsamifera***
280 mg/kg		6135.67 ± 220.87	25.00 ± 1.53	0.9996 ± 0.0001	28.33 ± 3.63	18.58 ± 0.38	0.9953 ± 0.0003
380 mg/kg		7448.00 ± 1121.96	27.33 ± 0.33	0.9994 ± 0.0002	31.22 ± 0.83	18.61 ± 1.59	0.9953 ± 0.0003
9040 mg/kg		6949.67 ± 1305.29	29.00 ± 2.33	0.9991 ± 0.0000	47.00 ± 9.87	19.48 ± 0.41	0.9953 ± 0.0007


**FIGURE 2:**
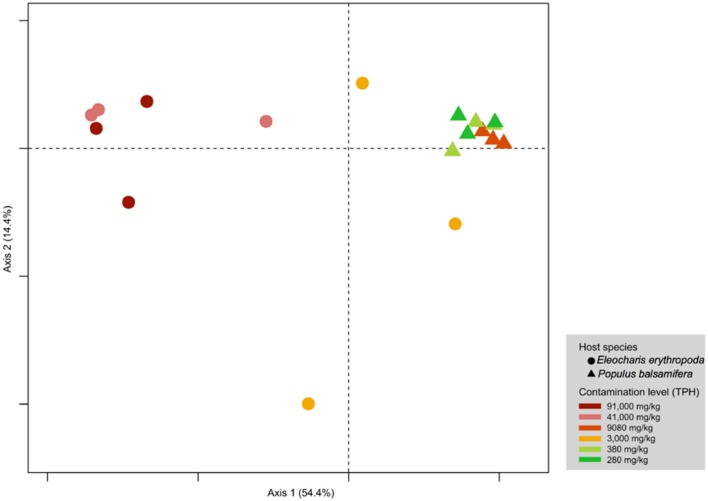
**Principal coordinate analysis of fungal endophytes of *E. erythropoda* and *P. balsamifera* roots.** The fungal communities were binned at phylotype resolution and were ordinated using PCoA based on Hellinger distance. Individual samples were colored based on the level of petroleum hydrocarbon contamination.

Principal coordinate analysis shows that the effect of the host identity on endophytic community structure was larger than contamination levels (**Figure 2**). PERMANOVA indicated a significant segregation of sites according to host species (*R*^2^ = 0.38994, *p* = 0.0001) and to contamination levels (*R*^2^ = 0.66753, *p* = 0.0001). However, the pairwise comparison did not support a significant clustering with respect to contamination levels.

**FIGURE 3:**
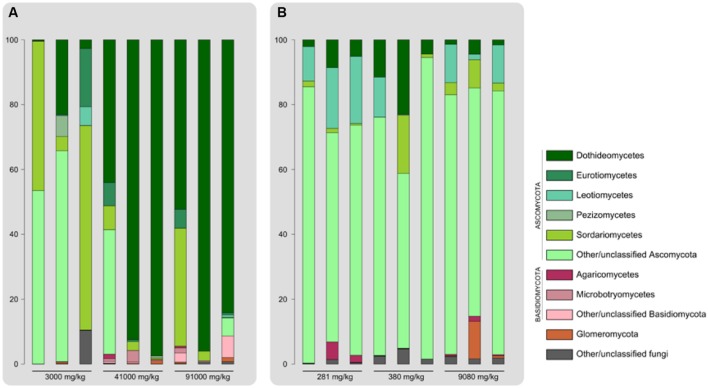
**Major classes of endophytic fungi recovered from roots of **(A)***E. erythropoda* and **(B)***P. balsamifera* Bar length is proportional to relative number of 454 reads**.

The fungal OTUs retrieved belonged to the phyla Ascomycota, Basidiomycota and Glomeromycota, as well as other unclassified fungal taxa. Endophytic communities appeared to be largely dominated by Ascomycetes in terms of relative read abundance across all contamination levels for both plant species. Taxonomic characterization of endophyte communities displayed distinct patterns in *E. erythropoda* and *P. balsamifera* (**Figure 3**). *E. erythropoda* samples harvested in the more contaminated soils (41000 and 91000 mg⋅kg^-1^ TPH) were dominated by Dothideomycetes, with a large proportion of sequences affiliated with this fungal class (between 44.00 and 97.46%), and most OTUs belonging to the order Pleosporales. Root samples from the lower contamination level of 3000 mg⋅kg^-1^ showed a higher relative abundance of Sordariomycetes (mainly from the order Hypocreales) and of unidentified Ascomycota.

On the other hand, *P. balsamifera* samples did not show significant shifts in the taxonomic composition, with unclassified Ascomycota largely dominating all samples. Leotiomycetes accounted between 10 and 20% of the total OTUs of the 280 mg.kg^-1^ of TPH samples. While Dothideomycetes dominated in *E. erythropoda* roots sampled in highly contaminated basins, unclassified Ascomycetes were dominant in *P. balsamifera* roots.

**FIGURE 4:**
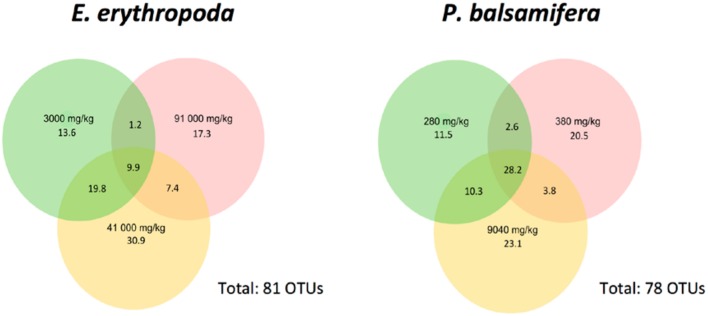
**Venn diagram showing the distribution of fungal OTUs among different concentrations of total petroleum hydrocarbon of *E. erythropoda* and *P. balsamifera*.** Total petroleum hydrocarbons (TPH) concentration is given in mg/Kg.

We compared OTUs shared between samples of each plant species (**Figure 4**). We found 9.9% of OTUs to be shared among plants growing in the different contamination levels in *E. erythropoda* while this value increased to 28.2% in *P. balsamifera* The 41000 mg⋅kg^-1^ TPH contamination level showed the highest percentage of unique OTUs in *E. erythropoda*, while the two other contamination levels (3000 and 91000 mg⋅kg^-1^ TPH) showed similar, but lower percentages of unique OTUs. However, the community composition was completely different between these contamination levels, with only 1.2% of OTUs shared. We also compared fungal isolates of culture-dependent community structure with amplicon sequencing datasets. We found that 454-sequencing did not reveal the same predominant genera of isolated fungal endophytes.

## Discussion

The community structure of soil microorganisms is influenced by vegetation and environmental conditions such as contamination levels (Ferrari et al., 2011; Bulgarelli et al., 2012; Abbai and Pillay, 2013; Bell et al., 2014; Chen et al., 2014). We investigated the community structure of endophytic fungi in two spontaneous plant species in relation to contamination levels using both high-throughput sequencing and isolation methods. Our results showed that contamination did not lead to significant changes in endophyte diversity measured with the Chao estimator for either plant species. Furthermore PCoA did not show significant variations in endophytic fungal community composition following changes in contamination levels. However, we could observe taxonomic shifts between *E. erythropoda* and *P. balsamifera* where *E. erythropoda* showed a greater abundance of Dothideomycetes in the 41000 mg.kg^-1^ and 91000 mg.kg^-1^ TPH samples which was not seen in the 3000 mg.kg^-1^ TPH samples.

While previous studies generally showed a negative correlation between the contamination levels and soil microbial diversity (Törneman et al., 2008; Ferrari et al., 2011; Bell et al., 2014), our results clearly showed that root endophytic fungi were not similarly affected. The cryptic nature of endophytic fungi, which live inside their plant host roots, compared to soil microorganisms may explain the differences in these results. Indeed, endophytic fungi may be more protected from environmental stresses and thus soil microorganisms might be more affected by stresses such as petroleum contamination. Furthermore, our study investigated endophytic fungal communities associated with spontaneous vegetation while Bell et al. (2014) investigated changes of introduced plant species. Plant species introduction can change soil biochemical properties, such as soil nutrient availability via root exudation, leading to the promotion of certain microorganisms in the rhizosphere (Van Breemen and Finzi, 1998; Reynolds et al., 2003; Bais et al., 2006).

The abundance of Dothideomycetes in *E. erythropoda* roots growing in high levels of petroleum hydrocarbon corroborates findings of Ferrari et al. (2011) and Bell et al. (2014) who found this group to be dominant in high hydrocarbon conditions. The genus *Alternaria* accounted for the majority of the Dothideomycetes sequences found in *E. erythropoda* roots. While generally described as a plant pathogen (Thomma, 2003), some *Alternaria* species do not exhibit pathogenic behavior and might help plants to deter pathogens through potential antimicrobial activity (Gu, 2009).

Our results suggest that the microbial community structure detected is highly dependent on the approach used. Isolation methods using different types of media and 454-sequencing did not reveal the same predominant genera. Similarly, Stefani et al. (2015) found distinct bacterial and fungal dominant taxa in hydrocarbon-contaminated soils with culture-dependent and culture-independent methods. Roche 454 sequencing offers a powerful molecular toolkit for studying microbial community structures in pooled samples. The main benefit of this approach is that it allows to process large number of samples and provides a large number of sequencing reads with relatively low cost. However, it has its pitfalls as reported by Tedersoo et al. (2010) where the authors compared 454-sequencing approach and the traditional method. Both methods yielded qualitatively similar results, but there were significant differences that affected the taxonomic view of the fungal community because of PCR biases and technical errors, therefore caution should be used for interpretation of results.

## Conclusion

The results of our study show that in some cases endophytic fungal community structure in plants is shaped by concentration of petroleum-hydrocarbon contamination. It also appears that culture-dependent and -independent methods do not promote the same strains, as the most abundant reads in 454 sequencing do not match with the most species recovered using isolation methods from samples taken from the same contamination levels. It is therefore important to better understand how plants recruit endophytic fungi and what their role is when inside the plant. This could potentially help develop newer and better strategies to improve phytoremediation using endophytic fungi. Improvement of such techniques could result in a better colonization of the soil by the plants with the help of endophytic fungi.

## Author Contributions

GB: Performed the experiments and wrote the paper. AR-B: Analyzed the data and wrote the paper. MS-A: Helped to design the experiments and wrote the paper. MH: Supervised, conceived, designed the experiments, and wrote the paper.

## Conflict of Interest Statement

The authors declare that the research was conducted in the absence of any commercial or financial relationships that could be construed as a potential conflict of interest.

## Footnotes

1 http://www.ncbi.nlm.nih.gov/.

2 http://www.R-project.org
